# Bilingual and Monolingual First Language Acquisition Experience Differentially Shapes Children’s Property Term Learning: Evidence from Behavioral and Neurophysiological Measures

**DOI:** 10.3390/brainsci9020040

**Published:** 2019-02-12

**Authors:** Agnes Groba, Annick De Houwer, Hellmuth Obrig, Sonja Rossi

**Affiliations:** 1 Institute of Special Education, University of Leipzig, Marschnerstr. 29 e, 04109 Leipzig, Germany; 2 Max Planck Institute for Human Cognitive and Brain Sciences, Stephanstraße 1a, 04103 Leipzig, Germany; obrig@cbs.mpg.de (H.O.); sonja.rossi@i-med.ac.at (S.R.); 3 Department of Linguistics, University of Erfurt, Nordhäuser Straße 63, 99089 Erfurt, Germany; annick.dehouwer@uni-erfurt.de; 4 Clinic for Cognitive Neurology, Medical Faculty, University of Leipzig, Liebigstraße 16, 04103 Leipzig, Germany; 5 Department for Hearing, Speech, and Voice Disorders, Medical University of Innsbruck, Anichstraße 35, A-6020 Innsbruck, Austria

**Keywords:** bilingual acquisition, monolingual acquisition, learning of property terms, adjectives, mutual exclusivity, whole object constraint, behavioral word learning task, fNIRS

## Abstract

Studies of novel noun learning show bilingual children rely less on the Mutual Exclusivity Constraint (MEC) for word learning than monolinguals. Shifting the focus to learning novel property terms (adjectives), the present study compared 3.5- and five-year-old bilingual and monolingual preschoolers’ adherence to the MEC. We found no bilingual-monolingual differences on a behavioral forced-choice task for the 3.5-year-olds, but five-year-old monolinguals adhered more to the MEC than bilinguals did. Older bilinguals adhered less to the MEC than younger ones, while there was no difference in MEC adherence between the younger and older monolinguals. In the 5-year-olds, we additionally acquired neurophysiological data using functional near-infrared spectroscopy (fNIRS) to allow for a first explorative look at potential neuronal underpinnings. The data show that, compared to bilinguals, monolinguals reveal higher activation over three brain regions (right frontal, left temporo-parietal, and left prefrontal) that may be involved in exploiting the MEC, building on conflict detection, inhibition, solution of a disjunction, and working memory processes. Taken together, our behavioral and neurophysiological findings reveal different paths towards novel property term learning depending on children’s language acquisition context.

## 1. Introduction

Early bilingualism shapes children’s language processing and language learning. For example, bilingually developing children are more sensitive to referential pragmatic deixis than their monolingual peers [1]. Therefore, when learning novel nouns [2,3,4] and adjectives [5], young bilingual children benefit more from deictic cues. They also outperform monolinguals on some measures of meta-syntactic awareness [6] and on their ability to integrate multiple cues in order to understand referential intent [7]. Moreover, general cognitive abilities such as inhibitory control [8,9], flexible switching [10,11], and memory flexibility [12] develop earlier in bilingual compared to monolingual children.

In the present study, we investigate how bilingual and monolingual first language acquisition experience shapes children’s property term learning. To reach this goal, we use both behavioral and neurophysiological measures.

### 1.1. The Whole Object Constraint, the Mutual Exclusivity Constraint, and Property Term Learning

Preschoolers generally tend to interpret novel words as nouns for whole objects, instead of mapping a novel word onto an object’s property [13]. This bias is known as the Whole Object Constraint (WOC) [14]. Depending on the languages(s) to be acquired, children have to learn word classes other than nouns [15]. Amongst these are adjectives, that is, terms to refer to properties. The learning of adjectives is a particularly challenging word learning task for young children (for a short review, see [5]). To tackle this challenge, novel adjective learning is facilitated by morphosyntactic markers [16,17,18,19] and descriptive gestures referring to object properties [5,17,20]. Here, we focus on another powerful cue.

Novel property term learning profits from the assumption that words have mutually exclusive meanings: the Mutual Exclusivity Constraint (MEC) [21]. Since each object should have only one name, monolingual children reject novel labels for known objects and search for alternative referents, a process termed disambiguation [22]. This allows monolingual children to indirectly select referents for novel words, in the absence of any explicit hint as to their intended meaning [23,24,25,26,27]. If the object in focus is familiar to the child and no unknown object is present in the communicative situation, children can search for an alternative referent *within* the familiar object. Candidates are a part of it, its substance, its surface, or other object properties [21]. For property term learning, the WOC (see above) must be overridden, rendering adjective acquisition more challenging than MEC-based indirect word learning for unfamiliar whole objects (nouns). In a task where novel labels were embedded in syntactic contexts ambiguous for adjectives or mass nouns, monolingual four-year-olds showed more property interpretations of novel words when an object was familiar than when it was unknown [27]. In another study with monolingual four-year-olds using a combination of cues, the MEC cue had a significant influence on property interpretations, but its efficiency was not tested in isolation [17]. Studies measuring the MEC contribution to property term learning in isolation are rare. Only two studies consider the effect of bilingual language exposure on MEC-based property term learning [28,29] (see Section 1.4).

### 1.2. Two Fundamentally Different Labeling Experiences: Bilingual and Monolingual First Language Acquisition

Children with regular bilingual input from birth (Bilingual First Language Acquisition) [30,31] learn what linguists describe as translation equivalents from very early on [32,33,34]. From the beginnings of word learning, they are used to the fact that objects may have two labels, namely one in each language. Already at 13 months, infants with regular bilingual exposure are surprised when speakers of different languages use the same label for the same object [35]. In order to be able to learn words in both languages, bilingual children are required and able to violate the mutual exclusivity assumption [34,36]. In this line of reasoning, the more translation equivalents bilingual children know, the less they use MEC-based disambiguation [37]. Conversely, infants (aged 17–18 months) with relatively few translation equivalents in comprehension are more likely to accomplish MEC-based disambiguation, indicating that the extent of bilingual word learning inversely modulates the use of the MEC. Therefore, bilinguals may have a propensity towards accepting second labels even within a single language [38]. 

In contrast to bilingual children, children with monolingual input from birth (Monolingual First Language Acquisition) [39] have been used to hearing mainly one word for the same thing. Thus, their language input coincides with mutual exclusivity assumptions and they expect speakers to share the same conventional word knowledge [40], even if they speak different languages [36]. Notably, monolingual children can also overcome the MEC: They may accept different labels for one and the same object if they are presented by two different speakers [41,42,43,44]. However, having different speakers refer differently to the same kind of object rarely happens in monolingual surroundings, whereas it is the rule in a bilingual input context [39].

### 1.3. Learning New Nouns: Differences and Similarities in MEC Adherence in Bilinguals and Monolinguals

The different labeling experiences of bilingual and monolingual children are reflected in research findings on learning new nouns. Eye-tracking studies show that infants with bilingual input from birth adhere less to the MEC than monolingual children: bilingual infants accept distinct labels for identical objects already at the age of nine to ten months, while their monolingual peers expect distinct labels to refer to different object kinds [45]. Similarly, a greater MEC-based disambiguation effect is found in monolingual infants between the ages of 17 to 22 months, with only marginal [46] or no MEC use in bilinguals [47]. In contrast to the latter studies, a longitudinal study of bilingual and monolingual children aged 18 and 24 months reports a similar propensity for MEC use [48]: both groups seemed to use the MEC by default in a simple disambiguation task. However, only the 24-month-old monolinguals succeeded in a retention task that required remembering the novel label learned in the disambiguation task. The authors conclude that only monolingual infants adopt the MEC as a reliable strategy for learning novel words, becoming stronger with age [48]. In contrast, bilingual infants might use the MEC as a default assumption for fast-mapping novel labels if no further information indicating the word’s referent is available. However, their everyday linguistic experience goes against the one-to-one-mapping [34,39] and dissuades them from establishing the MEC as a reliable strategy for word learning in the long term.

In a similar vein, research measuring preschoolers’ behavior in forced-choice word learning tasks showed higher MEC adherence in monolingual than bilingual children at age four [49]. Children with bilingual input from birth performed at chance level when they had to choose between mapping a novel word as a second label onto a familiar object or as a new label onto its unfamiliar part. Unlike their monolingual peers, who preferred mapping novel labels onto unknown parts of familiar objects, bilingual children therefore did not favor MEC-congruent responses. When mapping of the novel label onto the familiar object was cued, the bilingual but not the monolingual children performed above chance, showing that bilinguals were more willing to accept second labels than monolinguals. Developmental differences between three-, four-, and five-year-olds have been found for the learning of novel nouns in a complex disambiguation paradigm (on top of a forced-choice task children had to process information about the colors of the presented objects) [50]. At the age of three, ‘simultaneous bilingual’ children (who were judged “dual language learners” by schools and parents; it is not clear whether these children all experienced bilingual input from birth) were comparable to monolingual learners in the extent to which they exploited the MEC. However, at the age of five, the bilinguals’ novel noun interpretations (scoring at chance level) followed mutual exclusivity assumptions less often than the monolinguals’. Four-year-old bilingual and monolingual children’s differences in MEC-based object choices fell between the ones measured at three and five years of age [50]. A similar developmental trajectory was found for MEC use in three- and four-year-old versus five- and six-year-old bilingual and monolingual children [51,52]. The MEC-based disambiguation effect was more pronounced in the older monolingual than bilingual children, while no difference emerged amongst the younger age groups. The older monolinguals outperformed the younger ones [51,52]. This suggests a developmental increase in the disambiguation effect for monolingual children. Bilinguals showed similar responses at both ages for the mapping of new labels onto unfamiliar whole objects [51]. However, when children had to learn novel words for unknown parts of familiar objects (e.g., a piece of gold fabric added to a familiar plastic bottle) older bilinguals adhered less to the MEC than younger ones, suggesting a developmental decrease in MEC adherence in bilinguals [52]. Nevertheless, even five- and six-year-old bilinguals preferred mapping the new label onto an object’s unknown part than accepting a second label for the familiar whole object. Similarly, in Davidson et al. [51], the disambiguation rate was above chance for the older bilingual children, implying MEC-based processing. Another cross-sectional study also found evidence of MEC application in bilingual preschoolers, but no difference between four- and five-year old bilinguals [38]. The five-year old monolinguals, however, used the MEC more than the four-year old monolinguals. Besides looking at MEC application, this study also investigated the acceptance of lexical overlap. Bilingual five-year-olds outperformed four-year-olds in accepting lexical overlap, while monolingual peers performed similarly at both ages.

Summing up, in monolingual children, the MEC is accessible from very early on. The experience of highly frequent one-to-one-mappings fosters its development in a monolingual language learning context, leading to a developmental increase in monolinguals’ MEC adherence. In bilingual children, on the other hand, the everyday experience of multiple labeling leads to a developmental increase in accepting second labels for familiar objects and prevents bilingual children from strongly relying on the MEC in the learning of novel labels. Instead, bilingual children more strongly rely on other word learning cues such as pragmatic deixis [5]. If no alternative cues are present in the new word learning setting, bilingual children also seem to have access to the MEC, as a default assumption for word learning. It has been suggested that unreliability and unhelpfulness of a speaker can foster MEC adherence in three- and four-year-old bilingual preschoolers [53]. Application of the MEC in bilingual infants furthermore varies as a function of acquired translation equivalents [37].

In most of the studies on bilingual preschoolers aged three and older reviewed above, it is unclear whether the entire bilingual sample experienced bilingual input from birth. We focused only on studies where such an inclusion criterion was given [49] or very likely [4,38,47,50,51,52,53]. The latter studies may have included both children with bilingual input from birth (who have been used to hearing two labels for an object from the beginning) and early sequential bilingual children, who started off as monolinguals, hearing mainly one-on-one mappings. Precisely because of their different language input histories, it is essential to distinguish between these two bilingual groups [54,55]. With this caveat in mind, it still appears that bilingual preschoolers follow the MEC to a lesser extent than monolingual peers, and that bilingual preschoolers’ willingness to accept second labels becomes stronger with age. In the present study, we ask if the latter holds true for bilingual preschoolers with dual language input from birth in the special case of novel property term learning.

### 1.4. Learning New Property Terms: Differences and Similarities in MEC Adherence in Bilinguals and Monolinguals

The learning of novel property terms (adjectives) involves children having to contend with both the MEC and the WOC. A study of three-year-old early sequential bilingual children measured the effect of their bilingual language acquisition context on property term learning by including two cues aiding a property interpretation of the novel word: (i) objects were familiar to children, and (ii) novel words were morphosyntactically marked as adjectives [29]. Bilinguals showed better performance than monolingual peers, which is interpreted as evidence that bilingual children shift their attention more easily to a property of the object, thus overriding the WOC. This would indicate an MEC-congruent response that was more pronounced in the bilingual than the monolingual group, contrasting with the findings described above. An alternative explanation is that bilinguals have a generally higher sensitivity to morphosyntactic word form cues in order to identify intended word meanings than do monolinguals.

In contrast, an eye-tracking study with 17–18-month-old bilingual and monolingual infants confirms the findings of less MEC adherence in bilinguals than monolinguals for the learning of novel property terms [28]. In that study, bilinguals were exposed to English and at least one other language (time of first exposure to either is not reported). In the novel word learning experiment, children were familiarized with two known labels (“dog” and “cat”) and one novel label (“zabe”) through one-word-utterances without any sentential cues. Pictures of, for example, an orange dog, a purple cat, and a dog with another color (aqua) were paired with these labels. In the test phase, a purple dog (i.e., a category match item) and a cat colored in aqua (i.e., a property match item) were presented visually. The novel label was repeated twice, while children’s looking times were measured. As predicted, bilingual infants failed to adhere to the MEC and mapped “zabe” as a second label for a familiar category (e.g., dog). In contrast, monolinguals rejected “zabe” as referring to a familiar category and interpreted it as a property term for the color aqua. This was only observed when the novel label was repeated. No differences emerged when the novel label was used for the first time. The effect of a property interpretation in monolinguals was furthermore absent in another experimental condition. The authors conclude that monolingual infants’ ability to map novel words onto object properties is still fragile at this young age.

### 1.5. Neurophysiological Methods to Better Understand MEC Adherence in Young Children: Deductive Reasoning

To date, behavioral findings are inconclusive as to which word learning strategies bilingual and monolingual children use to learn property terms. Online MEC-based word learning processing has been investigated through several eye-tracking studies (Section 1.3), but, so far, neurophysiological studies shedding light on the underlying brain basis of cognitive processes involved in language learning are lacking. Neuroimaging methods could add more insight into which brain areas support different cognitive processes and could thus lead to a clearer understanding of the underlying neuronal mechanisms. 

Neurophysiological research has already explored deductive reasoning, which is possibly important for applying the MEC in novel property term learning. It has been proposed that deductive reasoning in the form of processing a disjunctive syllogism (A or B, not A, therefore B) underlies the disambiguation effect of the MEC [24] (p. 213). Crucial cognitive sub-processes associated with different neuronal correlates constitute the detection of a conflict (A or B), inhibition of a default interpretation (not A) and a logical deductive decision (therefore B). These steps impose substantial demands on working memory processes [56,57].

A complex neural network has been postulated to underlie deductive reasoning. It encompasses a left fronto-temporal region for heuristic processing based on world knowledge, a bilateral parietal region for logical-formal processing, a right lateral/dorsal prefrontal region for conflict detection, as well as left and right prefrontal regions for processing sure and unsure inferences, respectively [58] (p. 440). Moreover, left precentral, medial-frontal and posterior-parietal cortex have been discussed with regard to the processing of propositional syllogisms [59]. A disjunction constituting a special type of a propositional syllogism as described in the previous paragraph was reported to correlate with activations in the left precentral, the left inferior-frontal gyrus (Brodmann area, BA 44), and the left inferior-parietal cortex (BA 40) [60].

Further findings [61,62] suggest that inferior-frontal activations may be linked to verbal aspects of disjunctive processing, whereby purely deductive processes are associated with a medial-prefrontal (BA 8) and a left rostrolateral region (BA 10) [62]. Rodriguez-Moreno and Hirsch [61] propose a network recruiting left superior-frontal (BA 6, 8), right medial-frontal (BA 8) and bilateral parietal (BA 39, 40, 7) regions. A meta-analysis [63] links activations in the posterior part of the medial-frontal cortex to the detection of conflict and activations in the lateral prefrontal cortex (especially left-sided) to working memory processes. According to Van Overwalle [63], activations in the medial prefrontal cortex emerge from social-cognitive processing rather than deductive reasoning itself. In studies using functional near-infrared spectroscopy (fNIRS), activations in the right inferior frontal gyrus (IFG) were associated with inhibition of a default analysis after a conflict was detected between the familiar interpretation and the logical deduction [64,65]. Inhibitory control of a misleading strategy has also been investigated in an fMRI study with schoolchildren aged seven to eleven years showing right inferior frontal gyrus activation for inhibition [66]. Repetitive transcranial magnetic stimulation (rTMS) of the right IFG supported its prominent role in blocking a default analysis [67,68], and rTMS of the bilateral superior parietal lobule (SPL) hinted at its relevant function for analytic reasoning [68]. Inhibitory processes have not only been localized in right frontal regions [69], but also in left prefrontal structures, including the inferior frontal gyrus [70] (p. 3). Activations in the left prefrontal areas are supposed to be linked to the usage of inhibitory control in verbal working memory if resolving a task requires self-regulatory inner speech [70] (pp. 3–4).

Summing up, deductive reasoning is associated with a network engaging frontal and parietal regions [61]. Specifically, analytic reasoning has been associated with superior parietal regions [68], whereas propositional and disjunctive sub-processes seem to recruit posterior parts of the left frontal cortex [59,60] (see also for a review [71], p. 170). Inhibition of default strategies could be driven by the right inferior frontal cortex [58,64] or its left counterpart [70]. Further research in monolingual children and adults projects the neuronal correlates of inhibitory control onto frontal and prefrontal cortices [72,73,74]. The need for working memory support could be associated with activations in the left prefrontal cortex [63].

### 1.6. The Present Study

As reviewed above, both behavioral and eye-tracking measures have been used to examine the processing steps used by very young children in the application of the MEC. The present study goes a step further, additionally exploring cortical brain regions that may support MEC-based processing. It is the first study on MEC-based property term learning to implement a neurophysiological method, viz., fNIRS. Because of this study’s explorative nature, specific hypotheses are so far lacking and the interpretation of results must rely on more general cognitive capacities possibly underlying MEC-based processing as reviewed above (Section 1.5). fNIRS measures the vascular response non-invasively by applying near-infrared light. It allows for larger ecological validity than fMRI as children can comfortably sit on a chair in front of a monitor wearing a soft cap in which optodes are fixed. Furthermore, fNIRS is silent and hence ideal for experiments using auditory input. Despite these setting-related advantages, the topographical resolution of fNIRS is limited. Nevertheless, overall changes in brain regions can be assessed, thus allowing for a first inquiry into brain areas potentially relevant for the current research question. We compare fNIRS data with results from a behavioral forced-choice task in bilingual and monolingual five-year olds. Based on the literature reviewed in Section 1.5. We expect to find larger neural activations in monolingual than bilingual children in brain regions supporting deductive reasoning, conflict detection and inhibition as well as working memory, given that monolinguals usually are more willing to exploit the MEC than bilinguals.

For the behavioral measures, the present study investigates the role of the MEC in bilingual and monolingual children’s learning of novel property names in 3.5- and five-year-olds. We consider whether the developmental trajectory of MEC adherence found for the learning of novel nouns can be replicated for the learning of novel property terms. So far, there is only fragile evidence that MEC-based learning of property terms differs between bilingual (likely with input in two languages from birth) and monolingual children. Following Kandhadai et al. [28], we predict that bilingual children will map novel labels onto familiar whole objects with unfamiliar properties more often than monolingual children. The latter, in contrast, are expected to show more MEC-congruent responses by mapping the novel labels onto the objects’ unknown property. Following earlier findings for MEC application in the learning of novel nouns, this bilingual–monolingual difference is expected to be more pronounced in five- than 3.5-year-old children.

## 2. Materials and Methods 

This study was approved by the local ethics committee of the Medical Faculty of the University of Leipzig (project identification code: 262-1122082011). All parents gave their informed consent for inclusion before their children participated in the study.

### 2.1. Overview

We designed a novel property term learning task to collect behavioral data from 3.5- and five-year-old bilinguals and monolinguals. We additionally recorded neurophysiological data in a subgroup of the five-year-olds. All bilinguals had been regularly exposed to at least two languages from birth and most were tested in both a German and a Spanish version of the task. The monolinguals had been exposed to a single language from birth and were tested in that language (German).

Children were first familiarized with pictures of two identical known objects (e.g., two tables) whose surface was unfamiliar (e.g., a smudged blue–brown structure). During this familiarization, they heard a novel word that could grammatically be either a noun or a nominalized adjective. The familiarity of the objects and the lack of familiarity with the surface allowed for an application of the MEC: since the MEC predicts that an alternative label for a familiar object will be rejected, the label will be interpreted as referring to the unfamiliar surface. Children who follow the MEC will thus tend to show a property based response, while a category based response indicates acceptance of an alternative label for a known object. 

During familiarization, we recorded neurophysiological data for a subgroup of the five-year-olds. We used fNIRS, a method allowing for a rough localization of cortical activation during task performance [75,76], as detailed below (see Section 2.7 and following).

After familiarization, participants performed a behavioral forced-choice task: they chose between (i) another familiar object (e.g., a pot) with the same surface (e.g., smudged blue-brown structure), indicating an adjective interpretation, or (ii) the same object type (e.g., a table) with a different surface (e.g., a pattern of rectangles in different colors), indicating a noun We controlled the stimulus material for perceptual factors in order to minimize a potential confound interpretation due to visual variables (see Section 2.5).

The overall experimental design is similar to the one described in Groba et al. [5] but that study presented a word learning cue that differs from what we report on here.

### 2.2. Participants

Sixty-three bilingual and 57 monolingual children without any known developmental disorders participated in the study (45% girls). These were the same 120 children reported on by Groba et al. [5]. Children were living in one of two German cities, namely Leipzig and Berlin. The bilingual and monolingual groups each split up in a sample of 3.5-year-olds and five-year-olds. Thirty-one bilingual (mean age = 42.68 months; SD = 4.27, range: 2;11–4;3 years (years;months); 12 females) and 29 monolingual children (mean age = 41.28 months, SD = 3.68; range: 3;0–3;11 years; 11 females) formed the sample of 3.5-year-olds. The sample of five-year-olds split up in 32 bilingual (mean age = 59.81 months; SD = 6.05, range: 4;3–6;0 years; 16 females) and 28 monolingual (mean age = 60.54 months, SD = 3.06; range: 4;9–5;11 years; 15 females) children.

Our recruitment criterion for bilingual children was that they had been exposed to two languages (German and Spanish) from birth. The choice of German was dictated by the fact that the study was carried out in Germany and that thus a monolingual German-speaking comparison group could be found. Spanish was selected as the second language because of pre-existing contacts with the Spanish-speaking communities in Leipzig and Berlin, and because of the fact that the linguistic structure of Spanish allows a good comparison with German as far as adjective use is concerned (see Section 2.5). However, we did accept two children into the study who grew up with two languages from birth that were not German and Spanish, but Spanish and Catalan or Galician, and who started learning German only later (recruitment to fully fulfill our criterion had been difficult). Our recruitment criterion for the monolingual children was that they had grown up with only German input from birth. We made sure to include only monolingual children who had not had any regular contact with another language. 

Once bilinguals had been recruited, we asked parents to fill out questionnaires about global language input and use. All but two families with bilingual five-year-olds filled these in. Through these questionnaires, we learned that four of the 63 bilingual children had trilingual parental input from birth (German-Spanish, and Catalan, Farsi, Slovak, or Turkish; 3.5-year-olds: *n* = 3; five-year-olds: *n* = 1). One of these additionally heard some English at her daycare center. The 3.5-year-old with just Spanish and Catalan at home heard German and some English at his day care center. The five-year-old with just Spanish and Galician at home heard German at his preschool. A further 7 German-Spanish bilingual children additionally heard some English (*n* = 4) or French (*n* = 3), mostly in their child care center or preschool (3.5-year-olds: *n* = 3; five-year-olds: *n* = 4). 

The 59 children with German and Spanish input from birth that we had completed questionnaires for mostly heard German and Spanish through their parents (3.5-year-olds: *n* = 27; five-year-olds: *n* = 28). In 4/59 cases, both parents spoke just Spanish at home, while older siblings, neighbors or friends spoke German to the children (3.5-year-olds: *n* = 3; five-year-olds: *n* = 1). The Appendix (Table A1) lists additional information about the bilingual participants. 

The bilingual sample thus consists of only children with input in at least two languages from birth (59 two languages; 4 three languages). Sixty children were able to participate in both language versions of the experiment. Three of the 63 bilinguals did not know enough German and could only carry out the Spanish version (3.5-year-olds: *n* = 2; five-year-olds: *n* = 1); these were the two children with Spanish and Catalan or Galician input from birth and an additional child who had heard much more Spanish than German.

### 2.3. Behavioral Assessment

The experiment consisted of a short and a long version (see further Section 2.4). The short version was done without additional parallel fNIRS recording. The long version included fNIRS recording (see Section 2.7). Behavioral data were collected for all participants, either in a short or a long version. In principle, we aimed to use fNIRS for all the five-year-olds. Due to technical reasons, however, we were unable to obtain any fNIRS measurements for the 14 bilingual five-year-olds living in Berlin. Thus, we used the short behavioral assessment with all the 60 3.5-year-olds and with 14 bilingual five-year-olds. The long behavioral assessment was used with all the 28 monolingual and 18 bilingual five-year-olds (see further in this section for missing data).

Behavioral assessments of the short version were carried out in a quiet room of the children’s care centers or at their homes. Behavioral assessments of the long version were carried out at the Max-Planck-Institute for Human Cognitive and Brain Sciences in Leipzig. Half of the bilingual children started with the Spanish version of the experiment. On another day, within a 2-week period, they performed the experiment in the other language. Monolinguals performed only the German version.

All data sets of the 29 younger and 28 older monolinguals entered the analysis. For the bilinguals, 28 German and 29 Spanish datasets of the 31 younger children and 30 German and 30 Spanish datasets of the 32 older children were available for analysis (see Table 1). Missing data sets were due to (i) illness of 3 bilinguals in one session (exclusion for 3.5-year-olds in Spanish: *n* = 1; for five-year-olds in German: *n* = 1; for five-year-olds in Spanish: *n* = 1), (ii) insufficient German (3.5-year-olds: *n* = 1) or Spanish (3.5-year-olds: *n* = 1; five-year-olds: *n* = 1) knowledge, or (iii) not being raised with German from birth (see Section 2.2; 3.5-year-olds in German: *n* = 1; five-year-olds in German: *n* = 1). 

There were no differences in age for the bilingual and monolingual children who provided the German datasets (3.5-year-olds: *t*_(55)_ = 1.31, *p* = 0.197; five-year-olds: *t*_(44.58)_ = −0.94, *p* = 0.352).

### 2.4. Experimental Setting and Procedure

We designed a computer video game embedding the novel word learning task into a playful context. The game was programmed in Presentation^®^ (14.0, Neurobehavioral Systems, Berkely, CA, USA).

Children sat in front of a computer. The female experimenter communicated with children in the language of the respective experiment. In a brief introductory video clip, a female human-sounding ‘astronaut’ told the children there was going to be a party for aliens in outer space, and of course one needed to get presents to take to the party. Children were invited to ‘buy’ a present by selecting from two options on the touch screen (that was the goal of the game).

**Figure 1:**
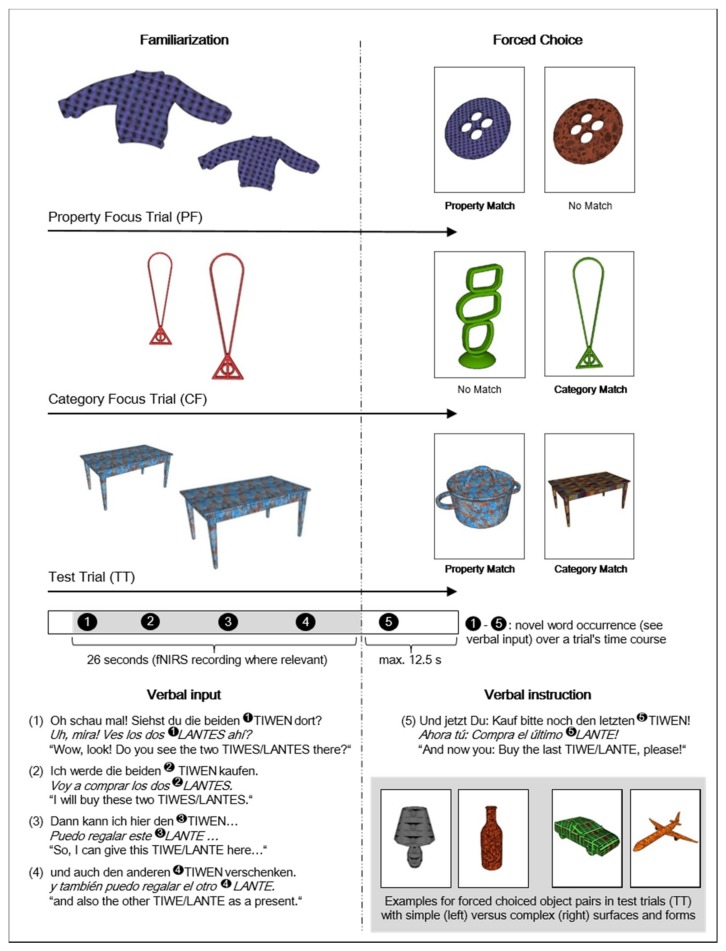
Examples for the familiarization phase (left) and the forced-choice task (right) in property focus trials (PF), category focus trials (CF), and test trials (TT) with corresponding verbal input (1–4) and verbal instruction (5). All trials were presented as films with the objects rotating around their axes in the familiarization phase. In the forced-choice task, the objects were presented as still images allowing for a touch response. Examples of stimuli that differed in complexity of surface and form (left pair: simple surfaces, *M* = 2.75 and *M* = 2.7, and forms, *M* = 2.0 and *M =* 1.43; right pair: complex surfaces, *M* = 3.95 and *M* = 3.95, and forms, *M* = 4.05 and *M* = 4.33) are shown on the bottom right. Influences of these potential confounds were attenuated by careful balancing across conditions (see text).

After this brief introduction, the experiment proper started. An experimental block consisted of 5 or 6 trials (see further in this section below. As shown in Figure 1, there were two types of trials: focus and test trials. Each trial consisted of a familiarization phase in which children saw two objects and heard the astronaut describe them through a novel word, saying that she was going to buy them. The familiarization phase was followed by a forced-choice task. Here, children were asked to ‘buy’ one of two objects on the screen by touching one of them (Figure 1, right). The question asking them to buy an object included the novel word used in the familiarization phase (Figure 1, verbal instruction). As explained below, there were three different kinds of objects of choice (property match, category match, no match object) depending on trial type. Regardless of which object children chose, each trial ended with a moving picture of a happy alien (6 versions). 

During the familiarization phase of each trial (Figure 1, left), rotating 3D-like pictures were presented of 2 exemplars of the same object, the Target Objects. They rotated in 5 sequences of different durations (1.5–4.5 s). In between each rotation, the objects stood still for 4 seconds and the verbal input was given. The novel word appeared 4 times in sentences as listed in Figure 1 (Verbal input). These sentences were syntactically ambiguous as to whether the novel word in them was a plural noun or an independently used adjective (see further Section 2.5).

We were interested in whether children applied a category or property interpretation of the novel words. To reduce bias and persistence to either one of these interpretations, each experimental block started with two focus trials. These trials directed children’s attention to either a category or a property interpretation. They were not test trials and did not enter the analysis. The experimental results were obtained in the test trials following the focus trials. The focus trials consisted of a property focus trial and a category focus trial, with counterbalanced order. 

In the familiarization phase of property focus trials (Figure 1, PF), children were shown two familiar target objects with unfamiliar surfaces that were described with a novel word. In the forced-choice task, they could choose between a PROPERTY MATCH and a NO MATCH object (Figure 1, right). PROPERTY MATCH objects matched the previously introduced target objects in surface but not in shape, i.e., they consisted of another familiar object with the same surface as the target objects. NO MATCH objects differed from the familiarized target objects in both property and category, that is, they consisted of a different familiar object with an unfamiliar surface that differed from the objects shown in the familiarization phase. In the forced-choice task of the property focus trials, the only logical choice was the PROPERTY MATCH object, highlighting an adjective interpretation of the novel word.

In the familiarization phase of category focus trials (Figure 1, CF), children were shown two unfamiliar target objects with known colors and plain surfaces that were described with a novel word. This should trigger a category-based interpretation of the novel word. For the forced-choice task part of the category focus trial, a NO MATCH object was paired with a CATEGORY MATCH object (Figure 1, right). CATEGORY MATCH objects and previously shown target objects had an identical shape but differed in surface, i.e., they were the same object with another color. NO MATCH objects again differed in both shape and surface from the target objects. In category focus trials, the CATEGORY MATCH object had to be chosen, highlighting a noun interpretation of the novel word.

The focus trials forced participants to follow either a property interpretation (→ adjective, PF) or a category interpretation (→ noun, CF) of the novel word. In both types of focus trials, the experimenter corrected children if they selected the No Match object. Half of the children started with focus trials supporting a property interpretation (PF). The other half started with category focus (CF) trials. 

After the two focus trials, a block of three or four test trials (TT) was presented. These were the novel word learning trials that entered the analysis. Test trials (Figure 1, TT) presented familiar objects with unknown surfaces, thus triggering the novel word’s property interpretation during the familiarization phase. The forced-choice tasks included one PROPERTY MATCH object and one CATEGORY MATCH object to test for MEC adherence. Selecting the PROPERTY MATCH object implied an adjective interpretation based on the MEC: children who reasoned that they already had a word for the familiar object would expect the novel word to refer to something else. The object’s property was then a likely candidate. Selecting the CATEGORY MATCH object indicated that the novel word was accepted as a second label for the known whole object, following the whole object constraint (WOC) instead of the MEC.

In both the property focus (PF) and the test trials (TT), familiar objects with unknown properties were used as targets, thus allowing for an MEC-based response (Figure 1, left). In contrast, novel objects with known properties (viz., colors) constituted the targets in the category focus (CF) trials, thus triggering a category based interpretation of the novel word. Because the objects were unknown to the children, the novel label could easily be mapped on the whole object without conflicting with the MEC (see Section 2.5 for more information on the objects used).

Left and right positions of the property and category match objects were balanced. 

Experiments were carried out with or without simultaneous fNIRS recording. When there was no fNIRS recording, children completed only one experimental block (Block 1; short version). Here, two focus trials were followed by four test trials (Table 2). The test trials were presented in randomized orders (24 different versions). When fNIRS was used, a second block of two more focus and three test trials in six different randomized orders followed. In the experiment’s long version (with fNIRS), the additional focus trials were added to prevent a perseveration or fixed response strategy that might have been adopted in the first block.

For the bilinguals, the experiment was carried out in both German and Spanish. The experimental design and instructions were identical in German and Spanish, but included two visually different astronaut actresses (1 German and 1 Spanish female adult) as well as different experimental stimuli for each language (see Section 2.5). In both language versions, objects and surfaces were similar in regard to visual salience, and novel words had similar phonological structures (see Section 2.5). As a third German-Spanish bilingual female adult spoke in both astronauts’ voices, identical voice quality in both versions was also ensured.

### 2.5. Stimulus Material 

The target objects in the property focus trials and test trials were familiar non-living objects with an unknown artificial surface (Figure 1, left). In order to facilitate novel adjective learning, two objects were used instead of just one [77]. ‘Living’ objects were excluded so as to avoid proper name interpretations [78,79]. Similarly, natural objects (e.g., vegetables) were not used, as their real surfaces would have conflicted with the artificial unknown surfaces. Familiarity of the objects was controlled for by selecting objects with an age of acquisition (AoA) that was mostly younger than the participants’ age. We used Spanish and German AoA datasets to help select objects [80,81]. Only objects with an AoA of 36 months or less were included, except for two forced-choice objects with slightly higher values (one in the German (37.09 months) and one in the Spanish (43 months) version). Children’s individual familiarity with the target objects was checked through a word-picture-matching task for the younger age group. Three Spanish trials for the bilingual 3.5-year-olds were excluded due to children’s lack of familiarity with the targets. 

We created 28 familiar object forms and 28 novel surfaces as object pictures for the test trials. We used a freeware version of the 3D modeling program SketchUp and the SketchUp Construction Library to extract and adapt or construct these stimuli and present them as films. To ensure that the familiar objects were clearly recognizable, we asked 21 German-speaking adults to name them. There was a 95% naming agreement for all stimuli used. One object had to be exchanged because it failed this criterion. Forms and surfaces were controlled for visual salience (surfaces) and shape complexity (forms), 2 factors that influence property term learning due to visual attraction [82,83]. Twenty German adults rated the visual salience of surfaces and 21 German adults the complexity of forms on a scale from 1 (low) to 5 (high). Surfaces with higher salience scores were combined with forms of higher complexity scores and vice versa. This should prevent children attending more to either surface or form due to the higher visual salience of either feature during familiarization. The forced-choice tasks consisted of property and category match objects of largely identical surface and form complexity (see for examples Figure 1, bottom right), to attenuate visual confounds during the decision process [84]. German and Spanish target stimuli were similar in surface salience and form complexity (surfaces: *t* = −0.254, *p* = 0.802; forms: *t* = 0.131, *p* = 0.896). Differences in indices of surface salience and object complexity within object pairings in the forced-choice task were also similar across the German and the Spanish versions (surfaces: *t* = 0.00, *p* = 1.0; forms: *t* = 1.09, *p* = 0.289).

The auditory input was recorded via Speech Record – AlgoRec, edited with Adobe Audition 1.0 (Adobe, San Jose, CA, USA), and monophonically presented via two loudspeakers. The verbal input consisted of four utterances followed by the verbal instruction each embedding the novel word (Figure 1, bottom). All German and Spanish sentential contexts could host either nominalized adjectives or nouns and were hence ambiguous with respect to a property (→ nominalized adjective) versus category (→ noun) interpretation. The novel words’ phonological structure also permitted either interpretation. They reflect a common structure of existing German and Spanish nouns (e.g., German: *Tasch-e*, “bag”; Spanish: *lech-e*, “milk”) or nominalized adjectives (e.g., German: *der Neu-e*, “the new one”; Spanish: *la grand-e*, “the big one”), they consisted of two syllables ending in a schwa for German (e.g., /ᴚe:fə/) and a mid-front vowel /e/ for Spanish (e.g., /nu:je/), and had a trochaic stress pattern. The novel words in the sentential contexts were inflected according to German (e.g., *Refe-n*) or Spanish (e.g., *nuye-s*) morphology. The definite articles used in the sentences matched the grammatical gender of the familiar objects and the names for the property match and category match objects were of the same grammatical gender. Thus, the definite article marking gender that they heard in the test question did not favor either of the two objects of choice. In both languages, about half of the test trials used feminine articles and the other half masculine ones. Feminine and masculine gender was balanced across trials.

Auditory and visual stimuli were merged using the video editing software Final Cut Pro (Apple Inc., Cupertino, CA, USA). The experimental game was completed by inserting the instructions of the videotaped ‘astronauts’ and the animated cartoons of happy aliens purchased from iStockphoto (Calgary, Canada). 

### 2.6. Analysis of Behavioral Data

As outlined in the introduction, preschool children are expected to show a preference for category match objects in novel word learning, due to their whole object bias (WOC). In the case of novel property term learning, MEC adherence indicates that the WOC is overridden (Section 2.4). We therefore used deviations from the WOC to rate children’s MEC adherence. We normalized category match choices to all responses (*n_category choices_*/*n_all trials_*). Four test trials per participant *(n_all trials_* = 4) entered the analysis, corresponding to the first block of test trials (whether in the short or long version). Lower values of *n_category choices_*/*n_all trials_* indicated a stronger deviation from the WOC, that is, more of an MEC-based choice.

Because the behavioral data was not normally distributed, we used linear mixed models (LMMs) instead of ANOVAs and non-parametric tests (Mann–Whitney) for post hoc testing. Two linear mixed models tested for differences in category choices between (i) GROUP (bilingual vs. monolingual, modeled as a fixed between-subjects factor) and (ii) LANGUAGE (German vs. Spanish, repeated within-group factor, covariance type: compound symmetry) (Note that the first comparison is between groups, while the latter is a within group comparison. An interaction between the two factors GROUP and LANGUAGE is therefore not possible). The factor AGE (3.5 vs. 5 years) was included as a fixed between-subjects factor in both LMMs. Type III sums of squares were used to test for fixed effects. Restricted maximum likelihood (REML) with 100 iterations was used for model estimations. IBM SPSS Statistics 24 – German version (Ehningen, Germany) served as data analysis tool.

### 2.7. Assessment of Cerebral Oxygenation Changes (fNIRS)

**Figure 2:**
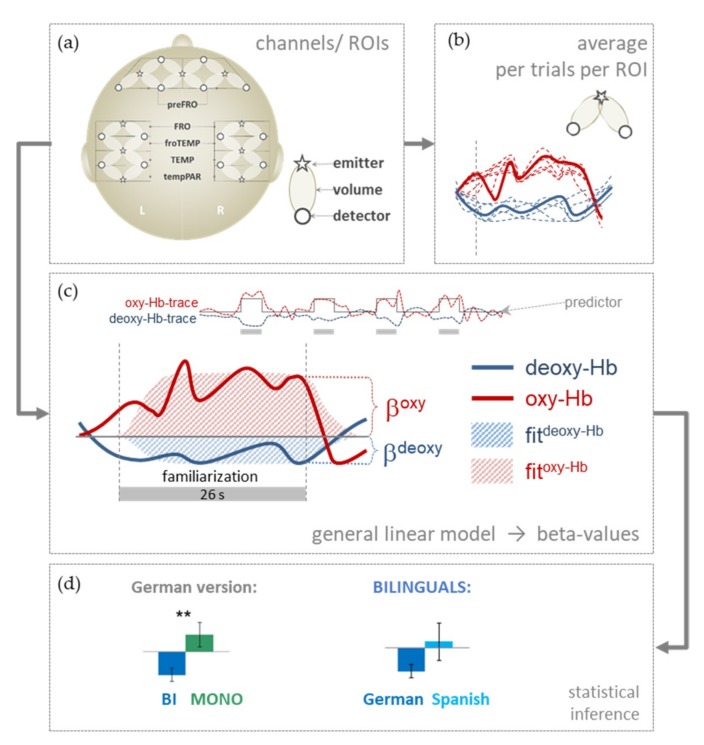
(**a**) setup of fNIRS measurements. Sampling volumes are determined by pairs of emitting (star) and detecting probes (circle). For statistical analyses regions of interest (ROIs) were defined over each hemisphere (left, L and right, R): prefrontal (preFRO), frontal (FRO), fronto-temporal (froTEMP), temporal (TEMP) and temporo-parietal (tempPAR). (**b**) Means across trials were calculated for each participant, channel, and ROI to judge the overall hemodynamic response. (**c**) For statistical analysis, a GLM-approach was used: a box-car-predictor of the stimulus period (here familiarization with novel word, presented 4 times) was convolved with the canonical hemodynamic response function and fit to the measured data (traces). This yields b-value as illustrated in the main plot. (**d**) The statistics were computed according to the contrasts of interest. Left graph: bilingual versus monolingual group (BI vs. MONO in the German version). Right graph*:* German versus Spanish version of the task for the bilingual group).

The 46 five-year-olds who were available for fNIRS recordings were tested at the Max-Planck-Institute for Human Cognitive and Brain Sciences in Leipzig. For the fNIRS recording, we used a dual wavelength continuous-wave system with 9 light emitters and 14 light detectors (NIRScout, NIRx Medizintechnik GmbH, Berlin, Germany/New York, NY, USA) covering bilateral prefrontal, frontal, temporal, and parietal areas based on 26 channels defined by all possible next-neighbor source-detector combinations (Figure 2a). The source-detector distance was approximately 2.5 cm. Probes were mounted using a modified EEG cap (Easy Cap, Herrsching, Germany). All 26 channels were used to define 10 regions of interest (ROIs), with 5 ROIs over each hemisphere: A prefrontal (preFRO), frontal (FRO), fronto-temporal (froTEMP), temporal (TEMP), and a temporo-parietal (tempPAR) region.

The fNIRS system supplies continuous readings (sampling rate 6.25 Hz) of changes in light attenuation at two wavelengths (760 and 850 nm) to be converted into concentration changes in oxygenated (oxy-Hb) and deoxygenated (deoxy-Hb) hemoglobin based on a modified Beer–Lambert approach [85]. According to the principles of neurovascular coupling, an increase in oxygenation is expected over an activated brain area [75,86]. Thus, increases in oxy-Hb and decreases in deoxy-Hb can be interpreted as markers of cerebral activation similar to other imaging techniques such as fMRI that are also based on hemodynamic response [87].

### 2.8. fNIRS Datasets

Eighteen bilingual and 28 monolingual five-year-olds underwent fNIRS measurements while performing the longer version of the experiment in German (no fNIRS for 14 bilingual children living in Berlin). The bilingual children also participated in the Spanish version. A total of 48 datasets (34 in German, of which 13 for bilingual and 21 for monolingual children; 14 in Spanish, all for bilingual children) entered the analysis. There were no differences in age (*t* = 0.29, *p* = 0.977) for the 13 bilinguals (mean age = 60.62 months; SD = 5.61, range: 55–72 months; 7 females) and 21 monolinguals (mean age = 60.57 months; SD = 3.30, range: 57–71 months; 11 females) who provided the 34 German fNIRS datasets.

Due to illness or exclusion, some data were not available. Exclusion of 16 datasets resulted from: cancelled appointments (2 German and 2 Spanish bilingual datasets), insufficient proficiency in Spanish (1 Spanish bilingual), not having German as language acquired from birth (1 German bilingual; see Section 2.2), technical problems (3 German bilinguals, 2 Spanish bilinguals, 5 monolinguals), insufficient data quality (2 monolinguals) due to low calibration values, excessive artifacts or the signal not showing heartbeat, thus indicating insufficient attachment.

### 2.9. Stimulation Period Analyzed by fNIRS

In vascular-based imaging techniques such as fNIRS event related designs are possible, although the sluggish vascular response peaks some 5–7 seconds after stimulus onset. However, since we were interested in the process of familiarization in the overall context, we modeled the whole familiarization phase as a predictor for the GLM-based approach (Figure 2c). The rationale is that applying the MEC in conjunction with a novel word is a process extending over the full presentation of the stimuli and is not limited to the relatively brief co-occurrence of the novel word and the familiar objects. Therefore, fNIRS-trials started with the first co-occurrence of a novel word and a familiar object. Familiarization lasted 26 s, including 3 more co-occurrences of the novel word (Figure 1). Analysis of the fNIRS recordings therefore included the period from 1s prior to children hearing the first novel word until 9 s after its last occurrence. Thus, the fNIRS epoch comprised 36 s for each stimulus. We analyzed the stimulus periods of nine trials per participant (Figure 2b). These stimuli were part of the two property focus and seven test trials of the experiment’s long version. We included the property focus trials because their familiarization phase presenting familiar objects with unknown surfaces was identical to the one used in test trials.

Interstimulus intervals between trials were jittered using films of three different lengths showing happy aliens (*M* = 7 s; range: 5–9 s). Variations in participants’ reaction times in response to the forced-choice task led to additional temporal jittering. Such jittering helps to attenuate effects of low-frequency background oscillations that do not directly reflect stimulus evoked neuronal activity [88].

### 2.10. Analysis of fNIRS-Data

We assessed the concentration changes of oxygenated (oxy-Hb) and deoxygenated hemoglobin (deoxy-Hb) in response to the stimuli (Figure 2c). There has been substantial debate about which of the two parameters is more robust. Additionally, some authors have postulated deviations from the typical adult response in children [89]. Therefore, we analyzed both parameters, i.e., increases in oxy-Hb and/or decreases in deoxy-Hb, separately [90].

Using a linear interpolation approach, sharp rises or falls suggesting motion artifacts were corrected channel-wise. This procedure used in a number of previous infant studies [91,92,93] requires visual inspection of every trial. In case a brisk, clearly non-physiological step in the NIRS-readings is detected, this step is marked and the data prior to and after the step are fitted replacing the artifactual step by a linear interpolation. After this procedure, data was low-pass filtered at 0.3 Hz to attenuate oxygenation changes that are related to heartbeat. After this filtering, we visually inspected all trials to enhance reliability of the artifact detection. Next, data were entered into a general linear model (GLM) yielding β-values for oxy-Hb and deoxy-Hb assuming a hemodynamic response function peaking at 5 s [94]. The resulting data (beta-values corresponding to µmolar changes; Figure 2c) represent the mean change in the two hemoglobins over the full trial length of 36 s compared to the (high-level) baseline. This ‘baseline’ includes both visual and auditory input. Therefore, changes in the two hemoglobins are also expected during this high-level baseline. High-level baselines may induce additional noise but are clearly preferable to ‘resting’ periods without any stimulation, especially in infant studies targeting cognitive tasks. Averages were computed for each channel in each participant (Figure 2b). Based on the variance of the data values higher than 7.0 µmol/L were classified as outliers and were subsequently excluded (<0.6% of the data). For further statistical analyses (Figure 2d), the data of different channels within each ROI was averaged (Figure 2b).

To test for differences between (i) GROUP (bilingual vs. monolingual, modeled as a between-subjects factor; Figure 2d) and (ii) LANGUAGE (German vs. Spanish, within-group factor; Figure 2d), we ran two separate Greenhouse–Geisser corrected ANOVAs. The factor Roi (10 ROIs; Figure 2a) was included as a within-subjects factor in both ANOVAs. ANOVAs were performed separately for oxy-Hb and deoxy-Hb data. The latter did not yield any statistically significant results (data not shown in the Results below).

The fNIRS analysis fundamentally follows the same principles as applied in Groba et al. [5]. 

## 3. Results

We predicted monolingual children to adhere more strongly to the MEC cue, resulting in a lower proportion of category choices compared to bilinguals. The difference between groups was expected to be more pronounced in the older age group, due to inverse developmental trajectories for bilinguals and monolinguals. For the bilinguals, no differences between German and Spanish experimental versions were expected.

### 3.1. Behavioral Data

#### 3.1.1. German Version: Effect of GROUP and AGE

**Figure 3:**
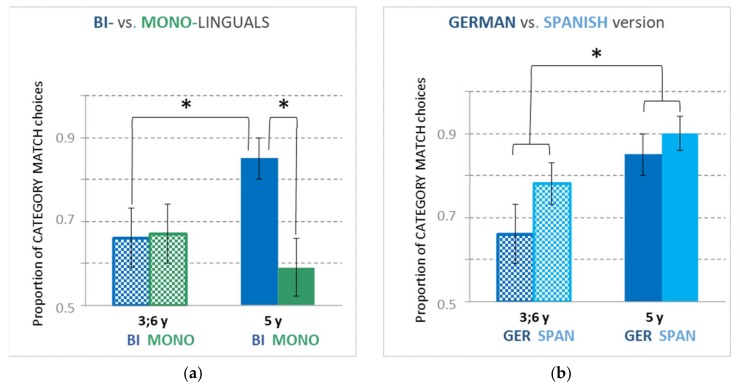
Behavioral data regarding the proportion of category match choices in the forced-choice task. Diagrams provide means and standard errors (+/−1) separately for 3.5-year-olds (checked bars, 3;6 y) and five-year-olds (full bars). Significant differences are indicated (*, *p* ≤ 0.05). (**a**) data of the German version for bilingual (dark blue, BI) compared to monolingual (green, MONO) children; (**b**) data of the bilingual children for the German (dark blue, GER) compared to the Spanish (light blue, SPAN) version.

All children revealed a category match bias, irrespective of group, age or language (Figure 3).

The main effect of GROUP showed a trend (*F*_(1, 111)_ = 3.44, *p* = 0.066), indicating that, compared to monolinguals (*M* = 0.63, SD = 0.39), bilinguals (*M* = 0.76, SD = 0.34) more often chose the category match object. Thus, bilingual children tended to violate the MEC more frequently.

There was no significant main effect of AGE (*F*_(1, 111)_ = 0.62, *p* = 0.431), but the interaction of GROUP × AGE was significant (*F*_(1, 111)_ = 4.11, *p* = 0.045). Contrary to our predictions, post hoc testing revealed that at the age of 3.5 bilinguals (*M* = 0.66, SD = 0.39, *Mdn* = 0.75) and monolinguals (*M* = 0.67, SD = 0.37, *Mdn* = 0.75) did not differ in the proportion of category choices (*U* = 395.50, *Z* = −0.18, *p* = 0.859, *r* = −0.02). At the age of 5, though, in line with our predictions, bilinguals (*M* = 0.85, SD = 0.27, *Mdn* = 1.00) chose the category match object more often (*U* = 277.00, *Z* = −2.45, *p* = 0.014, *r* = −0.32) than did monolinguals (*M* = 0.59, SD = 0.41, *Mdn* = 0.50). Interestingly, this effect was predominantly driven by monolingual girls (*M* = 0.42, SD = 0.41, *Mdn* = 0.25), who made significantly fewer MEC-incongruent object category choices (*U* = 49.00, *Z* = −2.35, *p* = 0.019, *r* = −0.44) than monolingual boys (*M* = 0.79, SD = 0.32, *Mdn* = 1.00). In all other subgroups, girls and boys behaved similarly (at age 3.5: bilingual: *U* = 90.50, *Z* = −0.27, *p* = 0.788, *r* = −0.05, and monolingual: *U* = 90.00, *Z* = −0.43, *p* = 0.666, *r* = −0.08; at age 5: bilingual: *U* = 110.50, *Z* = −0.08, *p* = 0.940, *r* = −0.01).

Following the interaction effect, post hoc tests were also run for age comparisons amongst bilinguals and monolinguals. Bilingual 3.5-year-olds (*M* = 0.66, *SD* = 0.39, *Mdn* = 0.75) chose the category match object less often (*U* = 299.00, *Z* = −2.08, *p* = 0.038, *r* = −0.27) than the older bilingual children (*M* = 0.85, *SD* = 0.27, *Mdn* = 1.00), who were more willing to accept second labels for the familiar objects. No age effect was seen for the monolingual group (3.5-year-olds: *M* = 0.67, *SD* = 0.37, *Mdn* = 0.75; five-year-olds: *M* = 0.59, *SD* = 0.41, *Mdn* = 0.50; *U* = 362.00, *Z* = −0.74, *p* = 0.457, *r* = −0.10).

Figure 3a provides the bar plots for the proportion of category match choices for bilingual compared to monolingual children for the German version.

#### 3.1.2. Bilingual Children: Effect of LANGUAGE and AGE

For the bilinguals, an LMM (AGE × LANGUAGE) yielded a significant main effect for AGE (*F*_(1, 61.04)_ = 6.79, *p* = 0.012) and a trend for LANGUAGE (*F*_(1, 57.89)_ = 3.79, *p* = 0.057); the interaction was not significant (*F*_(1, 57.89)_ = 0.70, *p* = 0.405). Similarly to their performance in German (see Section 3.1.1), bilingual children therefore also showed an age effect across languages, with five-year-olds (*M* = 0.88, SD = 0.24, *Mdn* = 1.00) more often choosing the category match object than 3.5-year-olds (*M* = 0.72, SD = 0.34, *Mdn* = 0.75).

The trend for a statistical difference between German and Spanish was driven by slightly fewer category choices in German (*M* = 0.75, SD = 0.34, *Mdn* = 1.00) than in Spanish (*M* = 0.84, SD = 0.25, *Mdn* = 1.00), suggesting that bilingual children were adhering slightly less to the MEC in Spanish than in German.

As intended, experimental sequence effects were not significant: children beginning with the German version did not differ in results from those starting with the Spanish one, either for German (*U* = 406.50, *Z* = −0.21, *p* = 0.837, *r* = −0.03) or for Spanish (*U* = 368.50, *Z* = −1.16, *p* = 0.247, *r* = −0.15).

Figure 3b provides the bar plots for the proportion of category match choices for the German compared to the Spanish version for bilingual children.

### 3.2. fNIRS Data (Limited to Five-Year-Olds)

#### 3.2.1. German Version: Effect of GROUP and ROI

The main effect GROUP showed a strong trend with medium effect size (*F*_(1,32)_ = 4.04, *p* = 0.053, η*_p_*^2^ = 0.11) driven by larger activation in the monolinguals (*M* = 0.46, SD = 1.02) compared to the bilinguals (*M* = −0.19, SD = 0.70). The main effect ROI (*F*_(9, 288)_ = 1.31, *p* = 0.258, Greenhouse–Geisser corrected, η*_p_*^2^ = 0.04) and the interaction ROI × GROUP (*F*_(9, 288)_ = 1.12, *p* = 0.350, Greenhouse–Geisser corrected, η*_p_*^2^ = 0.03) were not significant. 

**Figure 4:**
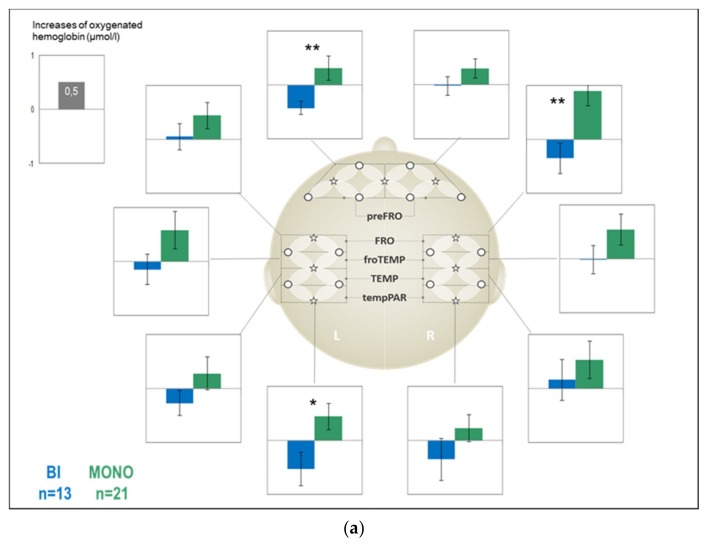

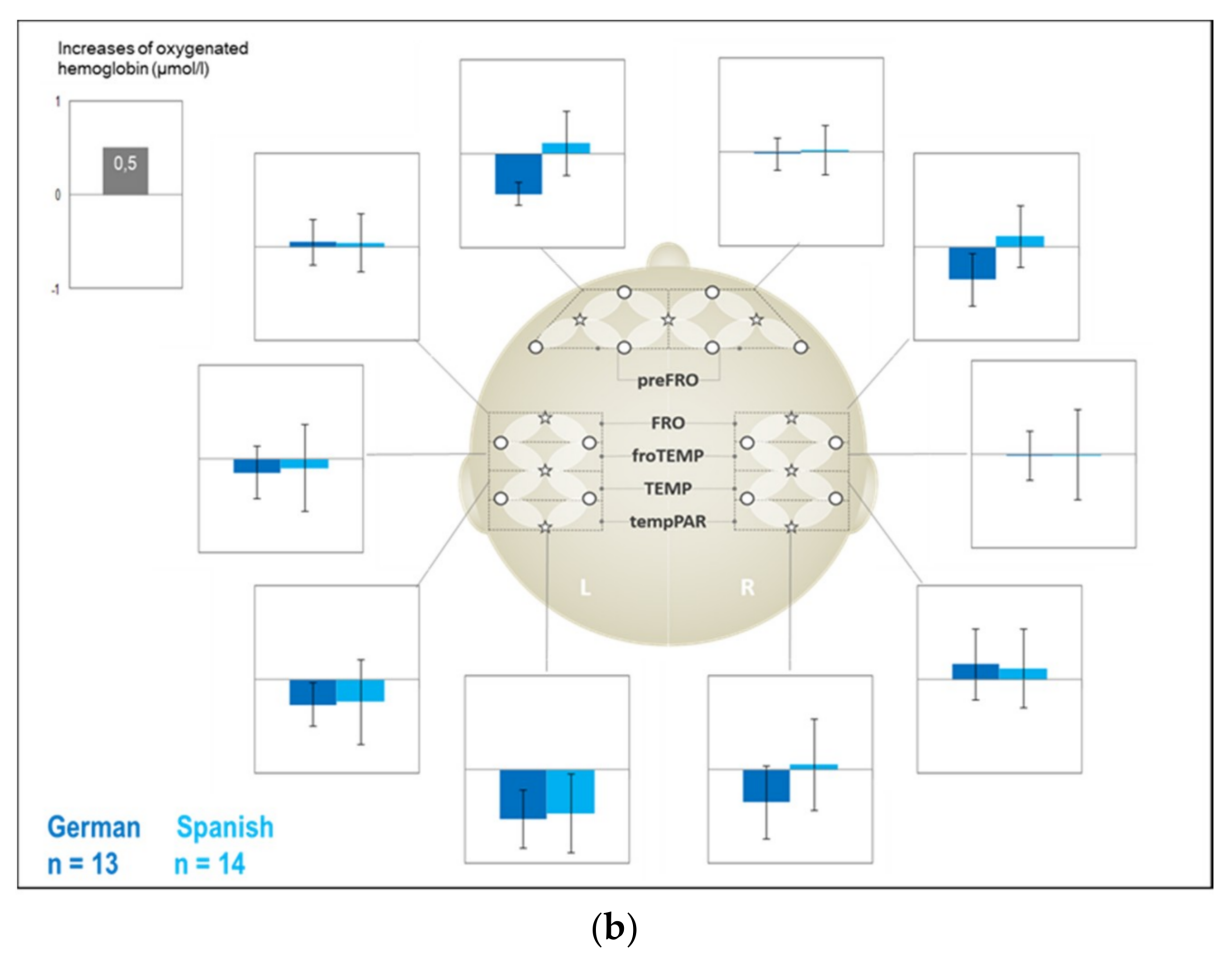
fNIRS results showing changes of oxygenated hemoglobin (µmol/l) during the familiarization phase in the five-year-olds. The diagrams show the groups’ means (bar plots) and standard errors (+/−1). Significant differences, found in the left (L) prefrontal (preFRO), right (R) frontal (FRO) and left temporo-parietal (tempPAR) region of interest, are indicated (*, *p* ≤ 0.05; **, *p* ≤ 0.01). (**a**) data of the German version for bilingual (dark blue, BI) compared to monolingual (green, MONO) children; (**b**) data of the bilingual children for the German (dark blue) compared to the Spanish (light blue) version.

Following the main effect of GROUP, visual inspection confirmed higher activation in the monolingual children over all ROIs. Although the interaction was not significant, *t*-tests showed significant group differences of large effect size favoring monolingual children in 3 ROIs: (i) left-preFRO (oxy-Hb↑; *t*_(28.35)_ = 2.87, *p* = 0.008, *d* = 1.01; monolingual: *M* = 0.31, SD = 1.06; bilingual: *M* = −0.43, SD = 0.42), (ii) right-FRO (oxy-Hb↑; *t*_(32)_ = 2.95, *p* = 0.006, *d* = 1.04; monolingual: *M* = 0.90, SD = 1.30; bilingual: *M* = −0.35, SD = 1.01), and (iii) left-tempPAR (oxy-Hb↑; *t*_(32)_ = 2.52, *p* = 0.017, *d* = 0.89; monolingual: *M* = 0.44, SD = 1.08; bilingual: *M* = −0.53, SD = 1.12). Figure 4a provides the bar plots for stimulus-locked oxygenation changes over all ROIs for bilingual compared to monolingual children for the German version.

#### 3.2.2. Bilingual Children: Effect of LANGUAGE and ROI

We performed an ANOVA for 13 German and 14 Spanish data sets in the bilingual sample. It revealed neither a main effect of ROI (*F*_(9, 90)_ = 1.65, *p* = 0.185, Greenhouse–Geisser corrected, η*_p_*^2^ = 0.14), nor of Language (*F*_(1, 10)_ = 0.55, *p* = 0.475, Greenhouse–Geisser corrected, η*_p_*^2^ = 0.05), nor their interaction (*F*_(9, 90)_ = 0.14, *p* = 0.949, Greenhouse–Geisser corrected, η*_p_*^2^ = 0.01). Figure 4b provides the bar plots for stimulus-locked oxygenation changes over all ROIs for the German compared to the Spanish version for the bilingual children.

### 3.3. Summary of Results

In summary, the behavioral results show that bilinguals chose the category match object more often than the monolinguals. This effect was most pronounced for the five-year-olds and indicates that bilingual children more often accepted a second label for familiar objects, while their monolingual peers adhered more strongly to the MEC. The fNIRS results for the five-year-olds showed that left prefrontal, right frontal and left temporo-parietal regions were more active during the familiarization with the novel word in monolingual compared to bilingual children.

While monolinguals performed similarly at both ages, the older bilingual children showed more category match choices across languages. They made slightly more category choices in Spanish than in German, but the fNIRS data did not show any language difference.

## 4. Discussion

Our behavioral and neurophysiological results support the hypothesis that monolingual children adhere more to the MEC than their bilingual peers when learning novel labels for an object’s properties. This extends earlier behavioral and eye-tracking studies showing a similar effect of language acquisition context for the easier task of learning novel nouns (Section 1.3). Our findings for the MEC-based learning of novel property terms extend recent eye-tracking results showing higher MEC adherence in monolingual than bilingual infants [28] to older children’s behavioral responses and cognitive processing. While we found an overall effect of bilingualism versus monolingualism across age groups, there was no overall effect of age. However, there was an interaction between language group and age: the bilingual–monolingual difference showed up selectively in the five-year-olds. This pattern of results is similar to results for novel noun learning reported by Aravind et al. [50], Davidson et al. [51], Davidson and Tell [52], and Kalashnikova et al. [38]. Intriguingly, in the complex property term learning task, five-year-old monolingual girls applied the MEC more often than their monolingual male peers. With monolingual boys being slightly older (*M* = 61.62, SD = 3.71) than monolingual girls (*M* = 59.60, SD = 2.06), age cannot explain the advantage in monolingual girls. Bialystok et al. [8] identified a similar effect for the easier task of learning novel object names based on the MEC in three-year-old monolingual girls, who outperformed both monolingual and bilingual boys. The authors suggest that girls are progressing more rapidly than boys in MEC development. One might add that, at early stages of lexical and grammatical development, monolingual girls have robustly been found to develop faster than boys [95]. This difference may lie at the basis of gender differences in MEC development in monolinguals, with more advanced linguistic skills leading to increased use of the MEC, which is an efficient word learning strategy in monolingual settings. 

Our data show no clear indications of MEC adherence in the bilingual children at the ages of 3.5 and five. Rather, the results speak for a developmental increase in the acceptance of second labels for whole objects, yielding a decrease in MEC adherence. This may be a result of bilinguals’ cumulative everyday experience with double labeling. Contrary to noun-learning paradigms [38,50,51,52], our study finds no strong evidence for developmental MEC strengthening in the monolingual group. However, the small (but non-significant) difference in category match choices between the younger and the older monolingual group supports the expected direction (more MEC adherence). Perhaps such a strengthening of MEC-guided property term learning only occurs in older monolingual children. For five-year-old monolinguals, the task may have been still too challenging, since it required overriding another powerful constraint (the WOC) in order to apply the MEC (see below in this section for further discussion). Because this complexity may also explain the near absence of MEC-based processing in the bilinguals, our data cannot settle the question of whether bilingual preschoolers start out using the MEC and diminish its usage with age. On the other hand, our data clearly show a developmental increase in the acceptance of second labels in bilinguals, leading to fewer MEC-congruent responses at the age of five compared to monolinguals. Similar to most previous studies comparing age groups [38,50,51,52], however, our study was cross-sectional rather than longitudinal, which may be necessary to disclose longitudinal trajectories [48].

As Byers-Heinlein and Werker [37] point out, the degree of MEC adherence in bilingual children is related to the number of translation equivalents in their bilingual lexicons, suggesting that bilingual children do not respond homogeneously when it comes to MEC-based processing. Given that there are likely even more opportunities for translation equivalents to exist when children know three languages [46], it follows that trilingual children might use a different approach to MEC-based processing than children acquiring just two languages. Byers-Heinlein and Werker [46] showed that bilingual and multilingual toddlers differed from each other in the extent to which they applied disambiguation strategies to interpret novel nouns. Our sample included a small (four) number of children with trilingual input from birth. This number is too small for statistical comparisons with the 59 children who heard just two languages from birth. Just four of the seven data points that the trilingual children supplied in the German and Spanish experiment exceeded the average proportions of category match responses compared to the whole group of bilingual children. However, taking together all the data points provided by children with experience with more than two languages, whether from birth or not, we find that in 17 of the relevant 21 cases their use of category match proportions was higher than the average for the entire bilingual group. Possibly, then, the inclusion of multilingual children in this study apart from strictly bilingual ones contributed to our finding of less MEC-based processing in the bilingual compared to the monolingual group, confirming, however, that the number of languages being acquired plays a role in the degree to which children are ready to accept lexical overlap. More systematic comparisons between children with strictly bilingual and trilingual input are needed to further explore the role of having heard two versus three languages from birth. 

Another reason for individual differences amongst bilinguals in MEC-based processing relates to the amount of input in each language [48]. A study of 94 four- to six-year-olds who grew up with English from birth and heard one non-English language either from birth or later in development, in very diverse circumstances, showed that relative amount and source of exposure to each language correlated with children’s acceptance of two labels for the same novel object [96]. The level of proficiency in the language production of four-year-old children with dual input from birth or with a chronologically second language may be a further modulating factor [97]. In addition, monolingual children’s degree of MEC adherence is modified by different factors such as age [25,98,99], vocabulary size [26], or level of particular word knowledge of the familiar objects as candidates for second label learning [57]. So far, we know little about the influence of such factors on novel property term learning through the MEC, but given our findings of an age related difference amongst bilinguals, it is likely that similar factors will also play a role for property term learning. Related to the issue of relative amount of input, the potential impact of another powerful key to word acquisition in general, namely statistical learning, should be mentioned. The relative frequency of one or the other label being used to refer to the same object (or property) may well modulate the strength through which the MEC has an impact on word learning. We are not aware of any study so far addressing the impact of a bi- or multilingual environment on statistical word learning in young children (see [100] for a recent review of the few studies on other aspects of statistical learning in bilinguals), but consider it an intriguing question to be addressed in future studies. 

A limitation of our study is the fact that we relied on only four test trials per behavioral assessment. One reason for having only four test trials for especially the younger children is the fact that the experiments reported here were part of a battery of experiments and tasks (another one is reported on in [5]), and that children would have been unable to concentrate for longer due to fatigue. In order to make comparisons across ages fully possible, we decided to also limit the five-year-old behavioral data to four test trials, even though for many of the five-year-olds we had three additional trials available. The fact that we have many behavioral assessments (174) somewhat compensates for the rather small number of individual trials.

To our knowledge, ours is the first study to combine behavioral and neurophysiological measures investigating MEC-adherence. As detailed below, we believe that our neuroimaging results support our behavioral findings of enhanced MEC use in monolinguals compared to bilinguals. Due to practical reasons, we did not record fNIRS in the younger age group. Eye-tracking studies have been able to identify bilingual–monolingual differences in MEC adherence in very young children [28,45,46,47,48] that were not apparent in behavioral studies with somewhat older children [38,50,51,52]. Therefore, it seems a promising approach to extend our fNIRS design to younger infants.

Regarding the interpretation of neurophysiological findings in the context of complex cognitive functions as required for the current task, we emphasize that differences in activation over specific areas of the cortex must not be over-interpreted, since differences in strategy or reliance on cues will rather be reflected in differences in network properties. However, the areas in which we find a statistically significant difference between groups and/or conditions have been discussed in the context of other more general cognitive abilities such as conflict detection and even deductive reasoning. The differences between activations undoubtedly confirm a difference in processing, while specific ascription to underlying cognitive functions is certainly somewhat speculative.

fNIRS probes covered cortical areas involved in the widely distributed neuronal network associated with deductive reasoning used to resolve a disjunctive syllogism. Our monolingual sample showed enhanced cortical activations especially over right frontal, left prefrontal, and left temporo-parietal regions of this network (The significant differences with large effect sizes between bilinguals and monolinguals over these three regions should nevertheless be treated cautiously as the ANOVA did not reveal a significant interaction effect between brain region (ROI) and GROUP). The right [64,65,66,67,68] or left [70] inferior frontal gyrus is considered to underlie the detection of a conflict and the inhibition of a default analysis. Therefore, larger responses in monolinguals could indicate that they perceived the occurrence of a novel label in the presence of a familiar object as a fundamental conflict. After detecting the conflict, the default strategy of mapping novel words on whole objects has to be inhibited. The monolingual fNIRS results therefore support claims about the prominent role of the right frontal cortex in conflict detection and in the inhibition of a default strategy [64,65]. In contrast to monolinguals, bilingual children showed less activation of right frontal regions, implying there was less MEC-based processing of the two labels for the familiar object as conflicting candidates. If bilinguals only perceived a minor conflict (or no conflict at all), they were not strongly triggered to inhibit the default strategy of mapping novel words on whole objects.

According to an MEC-based word learning process, after detecting a conflict between a novel label and a familiar object and after inhibiting the whole object bias, children should search for an alternative referent of the novel label, i.e., the object’s unknown property. Different cortical regions have been considered to support such a logical-formal processing step involving a disjunctive syllogism: a bilateral parietal region [58], superior parietal regions [68], and precentral inferior-frontal and inferior-parietal structures [59,60]. Van Overwalle [63], on the other hand, excludes the prefrontal regions as being responsible for deductive reasoning. Our results of larger involvement of the left temporo-parietal component of the deductive network in monolinguals than bilinguals might therefore be interpreted as a further indication of higher MEC use in the monolingual group.

Applying the MEC rests upon an elementary working memory support. The larger activations over the left prefrontal cortex in our fNIRS data for the monolingual group may reflect the involvement of MEC-triggered working memory processes. Similarly, Van Overwalle [63] links this brain region to working memory processes, and Houdé and Borst [70] attribute the left prefrontal region to the usage of inhibitory control in verbal working memory.

One further result that remains to be discussed is the influence of the particular languages on MEC adherence in the bilingual group. Bilingual children chose the category match object slightly more often in Spanish than in German. This result was only present as a statistical trend and was not supported by the fNIRS results. A tentative interpretation might be that the WOC is more prominent in Spanish than in German. This would hinder MEC application in Spanish more than in German. Gathercole and Min [101,102,103] found that Spanish syntax promotes children’s WOC adherence more than English syntax does. They explain this result by the fact that in Spanish it is generally possible to use any noun—also mass nouns—in a syntactic count noun context. Count nouns usually refer to individualized solid objects [101] with constant forms [104], supporting the assumptions of the WOC. Hence, Spanish speaking children can use the WOC in word learning for any kind of entity. In German, on the other hand, mass nouns are syntactically different from count nouns as many of them cannot be used as plurals. Mass nouns tend to refer to non-individualized substances (e.g., milk, water) [101] or materials changing their forms [104], which conflicts with WOC assumptions. The trend in our data regarding more WOC-based responses in Spanish than in German could reflect the slightly different count/mass noun distinction in both languages. A further explanation might be considered in this regard. Studies investigating sequential bilinguals found an impact of language proficiency on MEC adherence. Sequential bilinguals showed a reduction of MEC usage in their first language [105] and an equal or increased MEC usage in the second language [106]. Thus, less well developed language proficiency might lead to a preference for the MEC. As could be deduced from parental report, the children in our study generally spoke German better than Spanish. Given the findings for sequential bilinguals, one would thus expect the less well developed language (Spanish) to have given rise to increased MEC usage. However, an opposite tendency was in fact observable. The lack of a relative proficiency effect as found for sequential bilinguals may be a consequence of the fact that the bilinguals in our study were simultaneous bilinguals who had acquired both languages from birth. This brings us back to a language specific effect as proposed above, emphasizing a stronger reliance on the WOC in Spanish. 

Another point requiring discussion pertains to the generally non-preferred property term interpretation. Even monolingual five-year-olds did not show a preference for an MEC-based property interpretation of the novel label, although they showed such a preference more often than five-year-old bilinguals. Keeping all other linguistic (syntax, pragmatics) and visual (objects’ forms & surfaces) cues neutral, our experiment was constructed around the pure cue of object familiarity in the presence of an unknown surface. Other studies investigating the learning of novel property terms usually combine the cue of object familiarity with morphosyntactic marking of the novel word as an adjective [16,17,18,19,29]. Markman and Wachtel [27] also embedded the novel word in a sentential context that marked the novel label as an adjective or a mass noun, thus excluding a count noun interpretation. Furthermore, they chose substances of high salience instead of surfaces controlled for visual salience. The property match item of choice was not another object bearing the property but a chunk of the substance. Compared to our experiment, these aspects probably facilitated the task and explain why four-year-olds succeeded in interpreting the novel label as a property term in Markman and Wachtel’s [27] study. In contrast, the study by Kandhadai et al. [28] gave no morphosyntactic form class cues hinting at a property term interpretation. The property that the novel label in their study referred to was a particular color (aqua), i.e., a surface of presumably average salience. Furthermore, both stimuli of choice were familiar animate objects, i.e., pet animals. Similar to our study the MEC cue was hence presented in isolation, but in contrast with our study the MEC cue was enforced through naming another exemplar of the familiar object in advance. Thus, before hearing the novel label (e.g., “zabe”) while seeing an aqua dog, children were reminded that the familiar whole object they had seen before already had a name (i.e., *dog*). Monolingual 18-month-old infants looked significantly longer at the property match object than at the category match object. The authors conclude that monolingual infants seemed to treat the label as a property term. This effect, however, is fragile as it was only evident in one of two trials and in one of two experimental conditions. Nevertheless, why should 18-months-olds succeed in a task for which five-year-old children failed in our study? There are two possible explanations: firstly, reminding children of the known label of a familiar object could have given a strong hint, triggering MEC usage. Secondly, the differential method of eye-tracking versus task-based behavioral choices may explain the distinct results. Using eye-tracking allows the identification of MEC processes at a very young age, while behavioral effects show up only later in development. As we can see in our data, in the case of novel property term learning, the behavioral effect is still not present at the age of five. Perhaps at this age and even later in development a single cue is not sufficient to trigger a property interpretation of a novel word at the behavioral level. Rather, successful property term learning might require a combination of cues [17]. Besides the MEC, morphological and/or syntactic markers [18,19,107,108,109], prosodic features [110], and property- or object-inherent characteristics [79,82,83] might give important support. Pragmatic cues like descriptive gestures also aid property term learning [17,20] and, compared to their monolingual peers, bilingual five-year-olds show a heightened sensitivity to pragmatic cues fostering property term learning [5]. However, similarly to the heightened MEC adherence in monolinguals found in the present study, the solely pragmatic cue does not allow bilingual children to learn novel property terms on a behavioral level [5]. Combining the findings from the present study and those of Groba et al. [5], we suggest that (i) both bilingual and monolingual children need a combination of cues to successfully learn property terms in the ‘real’ world, and (ii) they weight possible learning cues in a hybrid learning context differently: While monolinguals rely more strongly on the MEC, bilinguals may favor pragmatic cues like descriptive gestures [5]. Future research should clarify which further cues are necessary to accomplish property term learning in bilingual versus monolingual children. Clinical intervention methods for children with difficulties in vocabulary acquisition could then be tailored to the particular needs of each population.

## 5. Conclusions

The present research corroborates the hypothesis of differential word learning strategies in bilingual compared to monolingual children. These strategies underlie differential developmental trajectories concerning adherence to the MEC. Specifically, we add to the research in investigating novel property term learning. Using fNIRS, our study allows for a first insight into the neuronal underpinnings of the novel property term learning process and differences between groups. Moreover, we constructed a word learning experiment around the single cue of object familiarity and carefully controlled for alternative cues. Thus, our results are more likely to truly reflect MEC-based processing in novel property term learning. This distinguishes our study from other research, which usually combines several learning cues. Another distinguishing feature of our study is that we carefully selected a homogeneous sample of bilingual children: they all grew up bilingually from birth, and most were acquiring the same set of languages, viz., German and Spanish. Testing both their languages is a further distinguishing feature.

At the age of five, bilingual children seemed to prefer a second label for a whole object to a higher degree than monolinguals, instead of rejecting a novel label for a familiar object and mapping it onto an objects’ unknown property. This propensity in bilingual children increased between 3.5 and five years of age. Conversely, monolingual five-year-olds followed the MEC more often than their bilingual peers. In addition, monolinguals revealed higher activations over three brain regions that are likely involved in exploiting the MEC: right frontal activations could reflect conflict detection between a familiar object and a novel label as well as inhibition of the default strategy (i.e., mapping the novel label onto a whole object). Temporo-parietal activations might stem from attempts to resolve the disjunction, i.e., from deciding for an alternative referent. The involvement of left prefrontal structures may be interpreted as an indication of working memory processes.

Similarly to the bilinguals, our monolingual sample did not show an overall behavioral preference for property-based interpretations of the novel word. We suggest that successful property term learning—which is a rather challenging task—builds upon a combination of learning cues with differential weighting in both bilingual and monolingual preschoolers. In addition to further learning cues, pragmatic cues could play a prominent role in bilingual children’s property term learning [5], while monolingual children continue to attend more to word learning principles like the MEC. We conclude that children fine-tune their word learning strategies in response to their particular language acquisition context.

## Figures and Tables

**Table 1:** Number of behavioral assessments available for analysis.

Group & Language	3.5-Year-Olds	Five-Year-Olds	All
bilingual, Spanish	29	30	59
bilingual, German	28	30	58
monolingual, German	29	28	57
All	86	88	174

**Table 2:** Trial types and their sequencing in the two experimental blocks.

Block	Trials
Block 1	**First 2 focus trials** 1 property focus trial
	1 category focus trial (order balanced across trials)
	**Followed by 4 test trials** (→ end of short version)
Block 2	**First 2 focus trials** 1 property focus trial
	1 category focus trial (order balanced across trials)
	**Followed by 3 test trials** (→ end of long version)

## Appendix A

**Table A1:** Information according to parental reports about the bilingual participants concerning their (i) German compared to Spanish input distribution during working days and (ii) on weekends, (iii) children’s verbal skills in German compared to Spanish and (iv) parents’ language choice with the children.

Input situation on working days	G >> S 3	G > S 46	G = S 7	G < S 4	G << S 0	missing 3
Input situation on weekends	G >> S 1	G > S 20	G = S 23	G < S 13	G << S 3	missing 3
Children’s reported verbal skills	G >> S 19	G > S 11	G = S 20	G < S 4	G << S 5	missing 4
Parental language use with children	one-G one-S 40	one-G one-G & S 6	one-S/G & S one-G & S 9	both-S others-G 4	one-S one-other 2	missing 2

G = German, S = Spanish, >> much more, > somewhat more, = equal, < somewhat less, << a lot less, one-G = one paent speaks mostly German, one-S = one parent speaks mostly Spanish, one-G & S = one parent speaks German and Spanish, both-S = both parents speak mostly Spanish, others-G = older siblings speak mostly German, one-other = one parent speaks other languages, missing = information not indicated by parents; note that the rows of information are to be read by themselves: they are not related to other rows.
